# The role of tissue maturity and mechanical state in controlling cell extrusion

**DOI:** 10.1016/j.gde.2021.09.003

**Published:** 2021-09-21

**Authors:** Teresa Zulueta-Coarasa, Jody Rosenblatt

**Affiliations:** The Randall Centre for Cell & Molecular Biophysics, Faculty of Life Sciences & Medicine, Schools of Basic & Medical Biosciences and Cancer & Pharmaceutical Sciences, King’s College London, United Kingdom

## Abstract

Epithelia remove dying or excess cells by extrusion, a process that seamlessly squeezes cells out of the layer without disrupting their barrier function. New studies shed light into the intricate relationship between extrusion, tissue mechanics, and development. They emphasize the importance of whole tissue-mechanics, rather than single cell-mechanics in controlling extrusion. Tissue compaction, stiffness, and cell–cell adhesion can impact the efficiency of cell extrusion and mechanisms that drive it, to adapt to different conditions during development or disease.

Epithelia cells work collectively together to provide a tight barrier to all the organs that they encase, often acting as the chief functional cells of each organ. Yet, cells turnover at high rates within these sheets by cell death and division that could potentially threaten this barrier. To maintain a healthy, functional tissue and barrier, excess, apoptotic, and often transformed cells are selectively eliminated by the process of extrusion. Extrusion can not only eliminate less fit cells, preserving the function of the tissue but can also act during development to help shape tissue and differentiate new cells. Epithelial cell extrusion ejects cells out of the layer without disrupting the epithelial barrier by collectively contracting an intercellular ring of actomyosin basolaterally [1]. Based on our current knowledge, live apical cell extrusion appears to promote most epithelial cell death in vertebrates [1–3]. However, during *Drosophila* development, most extrusion occurs basally into the tissue through activation of the apoptotic pathway [4]. In vertebrates, basal cell extrusion can drive developmental dedifferentiations [5–7] and can be co-opted by cancer cells to invade the underlying tissue [4].

## Tissue compression promotes cell extrusion

Epithelia are an intrinsic tensile fabric that are exquisitely responsive to stretch and compression forces. If there are too few cells, as after wound recovery, the epithelium will experience stretch, which can induce cell division [8–10]. If there are too many cells, or compressive forces during morphogenesis, cells can experience crowding, which induces live cell extrusion [2,3]. In this way, extrusion can limit compression forces and regulate epithelial cell density, to prevent carcinomas from developing [4]. In the developing pupal notum of *Drosophila*, tissue compaction activates the apoptotic pathway by mechanically down-regulating the epidermal growth factor receptor/extracellular signal regulated kinase (EGFR/ERK) pathway, which activates the pro-apoptotic protein Hid as well as cell extrusion [11••]. Alternatively, differential ERK activation can cause cell survival in stretched cells adjacent to the extruding cell as they close the gap. Unlike constitutive activation of ERK that causes cell extrusion [12], ERK pulses in surrounding cells inhibit caspase activation, protecting the epithelium from clusters of cells dying and extruding simultaneously, which would compromise its barrier function [13,14].

The stretch-activated ion channel (SAIC) Piezo1 controls crowding-induced live cell extrusion in cultured epithelial monolayers and developing zebrafish epidermis [3] and SAICs regulate live extrusion in mouse secondary palate fusion [15]. Although cell density typically increases by cell division or morphogenetic movements, wound repair in embryonic zebrafish can also cause crowding-induced extrusion [16]. Inhibiting SAICs during wound healing results in impaired cell removal and, instead, enhanced proliferation in crowded regions. Extrusion in this system could help eliminate damaged cells or those experiencing replicative stress [17] from rapid division required for tissue repair to ensure that substandard cells do not accumulate, following wounding.

Furthermore, exogeneous forces can also regulate crowding-dependent extrusion of endothelial cells during development. In early zebrafish dorsal aorta development, cyclic stretch generated by blood flow increases the dorsal aorta diameter and reduces endothelial cell extrusion [18••]. However, over time endothelial cells migrate ventrally towards the area where blood flow causes greater deformation, increasing cell density and causing cell extrusion. Importantly, a loss of function mutation in polycystic kidney disease 2 (*pkd2*), a mechanosensitive channel, increases cell extrusions in this ventral convergence zone, indicating that Pkd2 prevents endothelial cell extrusion in the dorsal aorta, in contrast to Piezo1’s role in promoting it in epithelia [3].

Another SAIC, TRPC1, promotes extrusion by polarizing neighbouring cells towards a cell fated to extrude. Here, a calcium wave initiates from Ras^V12^-transformed cells and travels to surrounding cells in both cultured MDCK monolayers and zebrafish embryonic epidermis [19]. TRPC1 then activates calcium waves in neighbouring cells to promote actin polymerization necessary to extrude the cell. It is not yet clear if TRPC1 is activated by stretch in response to the extruding cell contracting or by propagation of the initial calcium wave via gap-junction and IP3 receptor activation. Yet, this work highlights how a network of SAICs collaborate to successfully extrude a cell. While all these systems appear to use different signaling processes to trigger extrusion in response to compression, they all appear to act through calcium waves, suggesting a conserved pathway throughout species. Additionally, on a single cell level, compression tends to promote cell extrusion and death whereas stretch promotes cell migration and survival.

## Tissue-level intercellular tension regulates extrusion rates

Given that compression promotes cell extrusion, the stiffness of a given tissue can play an important role in regulating overall rates of extrusion. Recent studies suggest that increasing overall stiffness by expressing a constitutively active Rock kinase reduces cell extrusion in flies [20], whereas reducing it in fish embryos by inducing extensive apoptosis enhances extrusion rates [21,22]. By contrast, cells within the midline of *Drosophila* pupal abdomen preferentially extrude as epithelial cortical tension increases, measured by recoil from laser ablation [23••]. Here, caspase activation promotes cortical tension but is not sufficient to drive this change, as blocking caspase activity with P35 expression significantly reduces interfacial tension, but inducing apoptosis with *reaper* expression does not increase it. The findings that higher tissue tension promote apoptotic extrusion in some cases but decrease it in other cases suggest that other factors are at play.

Tissue-level contractile tension modulation is also important for live cell extrusion, which promotes most epithelial cell death in vertebrates. Recent work shows that high cortical tension within the monolayer prevents apical oncogenic cell extrusion of H-Ras^V12^ cells [24••]. Healthy cells can sense and extrude neighbouring H-Ras^V12^ cells from the epithelium through a cell-competition-driven process termed epithelial defence against cancer (EDAC) [25]. Here, depletion of caveolin-1 increases the phospholipid Ptdlns(4,5)P_2_ within the plasma membrane that, in turn, recruits the formin FMNL2 promoting actin polymerization and cell contractility. Notably, caveolar-dependent tension regulation is only necessary in the wild type cells neighbouring H-Ras^V12^ cells, rather than in the mutant cells themselves, showing the importance of differential regulation of tensions to allow extrusion.

## Different actomyosin structures mediate cell extrusion

**Figure 1:**
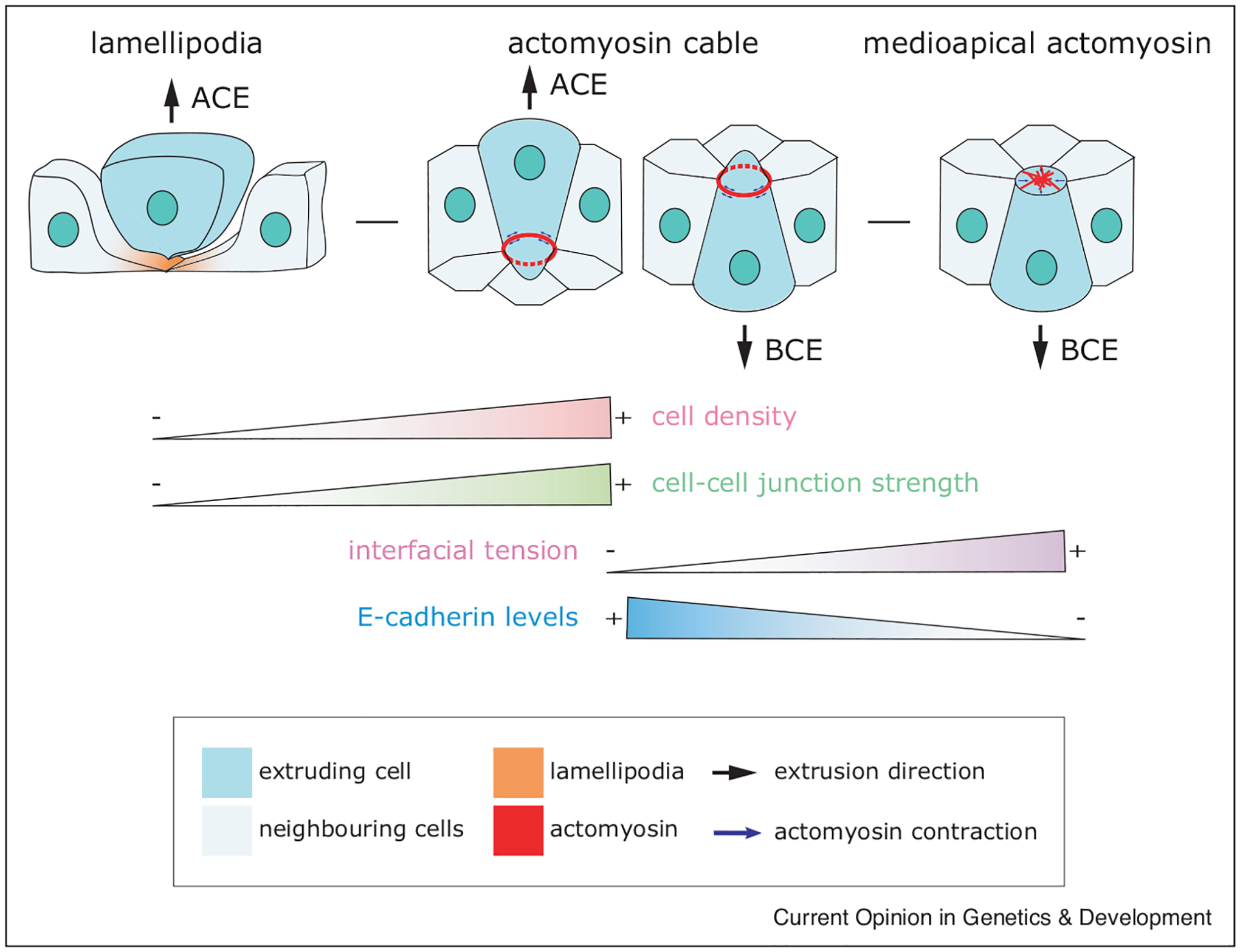
The mechanical properties of the tissue influence the extrusion mode. Apical cell extrusion (ACE) can be driven by basal lamellipodia crawling (left) or basal actomyosin cable contraction (centre-left). The switch between extrusion modes is mediated by cell density [27] and cell–cell junction strength [29••], with cells at higher densities and with stronger junctions extruding by cable contraction. Basal cell extrusion (BCE) can occur through the contraction of an apical cortical actomyosin ring (centre-right) or by medioapical actomyosin contraction (right). In *Drosophila* development the change in extrusion mode correlates with changes in cell adhesion and interfacial tension [23••], with cells extruding by medioapical contraction when tension increases, and E-cadherin levels decrease.

Depending on the state of the epithelium, actomyosin contraction can adapt to ensure a defective cell is eliminated by extrusion. Typically, cortical actomyosin first contracts apically within the extruding cell [1,26] followed by basolateral contraction of an intercellular network of actomyosin cables in the surrounding cells that act together to squeeze a cell out apically (Figure 1) [1]. Neighbouring cells can also send actin protrusions beneath the extruding cell to expel it and seal the epithelium, in conjunction with the contractile cable or in the absence of it (Figure 1) [27,28]. The mode of extrusion used depends on the strength of cell–cell versus cell-matrix adhesion [29••]. Interestingly, knocking-down a-catenin, the linker between the adherens junctions and the actin cortex, reduces cell–cell interfacial tension, promoting cell extrusion via basal protrusions (Figure 1). Conversely, expressing a form of a-catenin that constitutively recruits vinculin, reinforces actin at the cell cortex and shifts extrusion towards the actomyosin contractile mechanism. In this way, epithelial cell density can regulate the mode of cell extrusion in cultured cells, with cells grown in low densities, having higher attachments to matrix being extruded through basal protrusions, and those at higher densities with greater cell–cell than cell-matrix adhesions promoting canonical contractile extrusions (Figure 1) [27]. Because intact, mature epithelial tissues *in vivo* typically have stronger cell–cell adhesions, the basal cell protrusion extrusion mechanism may be reserved for situations where epithelia are not yet mature or are recovering from wounds.

In *Drosophila*, different extrusion mechanisms may be employed depending on the state of the tissue during development. In fly development, pulsed medioapical actomyosin contraction may help reduce the apical cell surface extrusion to promote extrusion (Figure 1) [30–32]. In pupae, two different mechanisms drive extrusion in separate abdominal epidermal cell populations [23••]. At early stages of pupal development, an extrusion mechanism that depends on cortical actomyosin contraction removes apoptotic epithelial cells at the border of histoblast nests [23••,^33^]. Later, however, epithelial cells near the fly midline extrude by combined contraction of cortical and medial actomyosin networks [23••]. The shift in which extrusion mechanisms is used could depend on whether the extruding cells are taller than the cells that replace them, differential signalling, or tissue maturity at different stages of development. Supporting the latter idea, embryonic wound repair in *Drosophila* occurs through medial actomyosin contraction within the wounded cell at early stages of development, but by a supracellular actomyosin cable at later stages [34]. How could tissue maturity affect the mode of epithelial gap closure? In the case of the fly pupal abdomen, cortical tension increases significantly as the larva develops [23••], while the levels of E-cadherin at cell–cell junctions decrease (Figure 1) [23••,35••]. Because interfacial tension depends on cell–cell adhesion and the contractile cortex, the mechanical context may dictate constriction modes during extrusion.

## Cell–cell junctional remodelling during extrusion

**Figure 2:**
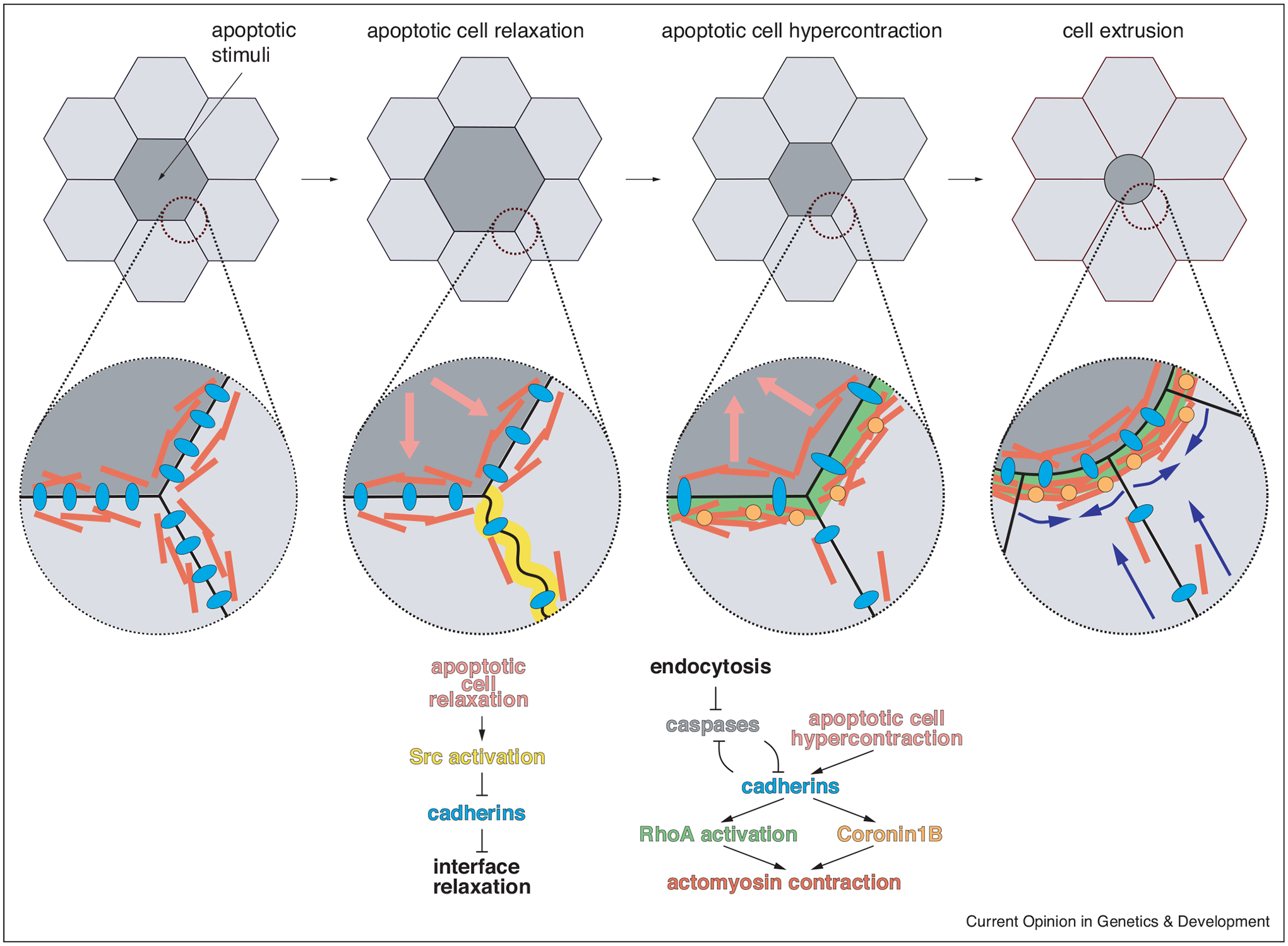
Adherens junctions remodelling and roles during apoptotic cell extrusion. Cells neighbouring and an extruding cell relax immediately, inducing the mechanosensitive activation of the Src family kinases at interfaces perpendicular to the extruding cell [37]. In turn, Src downregulates cadherins in these junctions promoting interface relaxation (centre-left). Then, contractility is increased in the apoptotic cell which promotes RhoA activation at the border between the apoptotic and neighbouring cells in an E-cadherin mechanosensitive manner [28] (centre-right). E-cadherin is also responsible to recruit Coronin1B to these interfaces, resulting in actin bundling [38]. Endocytosis reduces caspase activation, while caspases and E-cadherin mutually supress each other in a positive feedback loop [33,35••]. All together, these interactions result in competent cell extrusion, where the interfaces around the apoptotic cell efficiently contract, while the perpendicular ones can elongate permitting gap closure without losing barrier function (right, blue arrows).

Apart from their role in regulating tissue tension, adherens junctions are important to ensure efficient cell ejection and tissue integrity upon extrusion. Cell–cell and cell-matrix interactions must be tightly orchestrated to allow adhesion between new neighbors once a cell exits. During apoptotic cell extrusion in the fly pupa, the levels of adherens junction molecules decrease in a caspase-3-dependent manner, once actomyosin contraction begins (Figure 2) [33]. Cell–cell adhesion downregulation around the extruding cells promotes efficient cell removal, but it is not necessary for this process as embryos expressing the caspase inhibitor p35, which blocks E-cadherin downregulation, reduces but does not prevent extrusion. E-cadherin endocytosis in transformed cells and their neighbours is important for cancer cell removal via EDAC, suggesting that reducing cell–cell junctions helps enable extrusion [36]. In cells in culture and zebrafish embryos, transient tension release around extruding cells upon apoptotic injury activates Src kinases (Figure 2) [37]. In turn, Src activation reduces α-catenin and vinculin at cell interfaces perpendicular to the extruding cell to reduce interfacial tension and enable neighbouring cell elongation that will cover the space created by the cell’s exit.

While reducing cell–cell adhesions helps a cell detach, there is a growing body of evidence that E-cadherin is necessary for efficient extrusion in all systems. E-cadherin is required for extrusion, acting as a mechanotransductor to stimulate RhoA activity in the neighbouring cells in response to contractility within the extruding cell (Figure 2) [28]. E-cadherin is also important for F-actin organisation and contraction at the border between the extruding and the neighbouring cells [38] and to transmit forces and ensure barrier function in the surrounding tissue [39] (Figure 2). Similarly, delaminating cells maintain strong cell–cell adhesions with their neighbours until they get extruded from fly leg imaginal disk epithelia [40,41]. Interestingly, the classic epithelial to mesenchymal transcription factor Snail was recently found to promote basal cell extrusion from monolayers while retaining E-cadherin [42], a departure from the view that E-cadherins are transcriptionally downregulated before invasion and trans-differentiation. Finally, mathematical modelling suggests that the relative apicobasal levels of adhesion and contraction regulate the direction of extrusion [43].

How might cell extrusion simultaneously require cell–cell junctions and their downregulation? One possible way to interpret these seemingly opposing findings is that extrusion intrinsically needs cells connected within a monolayer or cells would just detach with no ability to preserve the functional barrier paramount to an epithelium. However, cells that are less adherent or weaker within the tightly woven fabric of a monolayer could make them more primed to extrude. Ultimately, cells with very tight contacts will have to loosen them, to enable their removal. Clearly more studies will help resolve this apparent contradiction.

One possible mechanism for controlling cell–cell junctions during extrusion is endocytosis. However, Hoshika *et al*. found, counterintuitively, that in *Drosophila* pupae, reduced endocytosis surprisingly leads to decreased E-cadherin and increases extrusion [35••]. The reduced E-cadherin presumably results from caspase-dependent degradation [33]. Here, because endocytosis reduces caspase activity, reduced endocytosis actually leads to reduced cadherin levels and increased caspase activation that would promote cell extrusion. Because E-cadherin and caspases mutually repress each other (Figure 2), both endocytosis and E-cadherin may play a protective role against apoptotic cell elimination in this system. Of course, it will be interesting to learn the role of E-cadherin regulation without the impact of caspases on live, apical extrusions that dominate in vertebrates.

Presumably other cell–cell junction components must be similarly regulated during extrusion. Septate junctions, the functional equivalent of tight junctions in *Drosophila*, remain intact during extrusion [33] as do desmosomal junctions [44]. Depletion of the desmosomal component desmoplakin results in disruption of the actomyosin ring around the extruding cell and failed extrusion. Down-regulation of the tight junction component Claudin6, promotes increased apical extrusion of cells in the *Xenopus* otic region reducing the otocyst size [45]. Thus, more work will determine how adherens and other junctional complexes are choreographed during both apical and basal cell extrusions.

## Shaping development

The connection between tissue mechanics and cell extrusion is important in several morphogenetic processes, not only to eliminate unfit or extraembryonic cells but also to generate new shapes and tissues. For example, recent work in the developing zebrafish heart shows that proliferation and overcrowding in the myocardium results in tension heterogeneities with some cardiomyocytes expressing higher levels of apical actomyosin [46]. As a result, the hypercontractile cardiomyocytes delaminate from the myocardium to help shape the tissue from a monolayer to a complex three-dimensional structure.

Similarly, cells in the dorsal pericardium experience overcrowding due to cell proliferation and migration towards the midline [47]. Consequently, cells are completely extruded from the tissue, creating a cluster of cells that will eventually give rise to the epicardium. Although most live extruding cells die due to lack of survival signals [3], these proepicardial cells are washed away by heartbeat-derived fluid flow to the pericardial cavity where they survive and contribute to morphogenesis. Notably, forces generated during extrusion can drive shape changes like the tissue folding of the *Drosophila* leg imaginal disks [40]. These results show that mechanical forces affect extrusion which, in turn, impact shape changes and cell fates.

Recent investigations have highlighted the impact of mechanics on extrusion during development. These findings have underscored the plastic nature of epithelia and how they cope with unwanted cells in different mechanical environments. Future work will need to address what regulates the mechanics that drive not only apoptotic cell extrusion but also live cell extrusion, which drives most epithelial cell death.
